# Structural insights into the molecular mechanism of phytoplasma immunodominant membrane protein

**DOI:** 10.1107/S2052252524003075

**Published:** 2024-04-24

**Authors:** Chang-Yi Liu, Han-Pin Cheng, Chan-Pin Lin, Yi-Ting Liao, Tzu-Ping Ko, Shin-Jen Lin, Shih-Shun Lin, Hao-Ching Wang

**Affiliations:** a The PhD Program for Translational Medicine, College of Medical Science and Technology, Taipei Medical University and Academia Sinica, Taipei, Taiwan; b Graduate Institute of Translational Medicine, College of Medical Science and Technology, Taipei Medical University, Taipei, Taiwan; c Institute of Biotechnology, National Taiwan University, Taipei, Taiwan; d Department of Plant Pathology and Microbiology, National Taiwan University, Taipei, Taiwan; e Institute of Biological Chemistry, Academia Sinica, Taipei, Taiwan; f International Center for the Scientific Development of Shrimp Aquaculture, National Cheng Kung University, Tainan, Taiwan; g Agricultural Biotechnology Research Center, Academia Sinica, Taipei, Taiwan; h Center of Biotechnology, National Taiwan University of Science and Technology, Taipei, Taiwan; Chinese Academy of Sciences, China

**Keywords:** phytoplasma, immunodominant membrane proteins, α-helix bundles, actin-binding proteins, protein structure, X-ray crystallography

## Abstract

Immunodominant membrane protein (IMP) is a prevalent membrane protein in phytoplasma and has been confirmed to be an F-actin-binding protein. However, the intricate molecular mechanisms that govern the function of IMP require further elucidation. In this study, the X-ray crystallographic structure of IMP was determined and insights into its interaction with plant actin are provided. A comparative analysis with other proteins demonstrates that IMP shares structural homology with talin rod domain-containing protein 1 (TLNRD1), which also functions as an F-actin-binding protein. Subsequent molecular-docking studies of IMP and F-actin reveal that they possess complementary surfaces, suggesting a stable interaction. The low potential energy and high confidence score of the IMP–F-actin binding model indicate stable binding. Additionally, by employing immunoprecipitation and mass spectrometry, it was discovered that IMP serves as an interaction partner for the phytoplasmal effector causing phyllody 1 (PHYL1). It was then shown that both IMP and PHYL1 are highly expressed in the S2 stage of peanut witches’ broom phytoplasma-infected *Catharanthus roseus*. The association between IMP and PHYL1 is substantiated through *in vivo* immunoprecipitation, an *in vitro* cross-linking assay and molecular-docking analysis. Collectively, these findings expand the current understanding of IMP interactions and enhance the comprehension of the interaction of IMP with plant F-actin. They also unveil a novel interaction pathway that may influence phytoplasma pathogenicity and host plant responses related to PHYL1. This discovery could pave the way for the development of new strategies to overcome phytoplasma-related plant diseases.

## Abstract 2

The first crystal structure of phytoplasma immunodominant membrane protein is reported and structural analysis revealed its potential for actin binding.

## 1. Introduction

Phytoplasmas are cell-wall-free bacteria that inhabit the phloem tissue of plants. They are transmitted by leafhoppers, plant­hoppers and psyllids of the order *Hemiptera* (Hogenhout *et al.*, 2008 ▸; Maejima, Iwai *et al.*, 2014 ▸). Phytoplasma infection causes abnormal plant development, including witches’ broom, dwarfism, fatal yellowing of leaves or phyllody (Namba, 2019 ▸). As phytoplasma infection is harmful to many economic crops, such as apples (Seemüller *et al.*, 2010 ▸), grapevines (Constable, 2009 ▸), cabbages (Lee *et al.*, 2000 ▸), coconut palms (Bonnot *et al.*, 2010 ▸), tomatoes (Ding *et al.*, 2013 ▸) and other crops (Lee *et al.*, 2000 ▸), but the underlying mechanism remains unclear, it is important to understand the pathogenesis of this pathogen in order to discover methods of prevention and treatment.

Phytoplasma immunodominant membrane protein (IMP) is a highly abundant and variable protein found on the surface of phytoplasma cells with a transmembrane region in its N-terminus (Shen & Lin, 1993 ▸; Yu *et al.*, 1998 ▸; Rossi *et al.*, 2019 ▸). IMP is involved in interactions between phytoplasmas and their hosts: both plants and insects (Wang *et al.*, 2023 ▸). It is considered to play a crucial role in the transmission and pathogenicity of these bacteria. IMP has been reported to be a useful molecular marker for the identification and classification of phytoplasmas, as it reflects the genetic diversity and evolutionary history of the different strains (Kakizawa *et al.*, 2009 ▸). Later, IMP was confirmed to interact with cyto­skeleton proteins, including F-actin and tubulin, and transgenic plants expressing IMP or the IMP ectodomain showed resistance to viral or bacterial infection (Boonrod *et al.*, 2012 ▸). IMP co-localizes with α-tubulin near the cell membrane, suggesting a relationship, and enhances the transmission efficiency of the phytoplasma (Ding *et al.*, 2022 ▸). Therefore, IMP is considered to be a potent target for the development of plant resistance strategies against phytoplasma infections. In this study, we determined the crystal structure of IMP, and structural comparison showed that IMP appears to have a similar conformation as the F-actin-binding protein talin rod domain-containing protein 1 (TLNRD1; Cowell *et al.*, 2021 ▸). The high structural similarity between IMP and TLNRD1 further suggests that IMP is an F-actin-binding protein. Moreover, by using proteomic approaches, we also identified IMP as a partner that interacts with the important phytoplasmal pathogenic factor phytoplasmal effector causing phyllody 1 (PHYL1). We therefore used a protein–protein docking method to investigate the different binding modes of IMP, TLNRD1, PHYL1 and F-actin. The docking results for IMP and TLNRD1 showed that these two proteins target the same location of F-actin with reliable docking and confidence scores. Interestingly, the proposed IMP–PHYL1 binding model also yielded similar results in a docking analysis with F-actin. This suggests that PHYL1 is also involved in the regulation of IMP binding to F-actin. Taken together, our observations could further extend our understanding of the functional roles of IMP in phytoplasma pathogenesis.

## 2. Materials and methods

### 2.1. Recombinant protein purification and antiserum production

The immunodominant membrane protein (IMP) used in this research was obtained from *Crotalaria* witches’ broom phytoplasma with deletion of the transmembrane domain (Accession No. AFK64762.1). The codon-optimized IMP gene (without the transmembrane domain; amino acids 33–172) was synthesized and cloned into the pET-28a expression vector by GenScript. Recombinant protein was generated in *Escherichia coli* strain BL21 cells. *E. coli* cells containing pET28a-IMP plasmid were cultured in 3 l LLB medium at 37°C for 3 h to an OD_600_ of 0.4. 1 m*M* isopropyl β-d-1-thiogalactopyranoside (IPTG) was then added for recombinant protein induction at 16°C overnight. The *E. coli* cells were collected and suspended in binding buffer (20 m*M* Tris base pH 8.0, 500 m*M* NaCl, 20 m*M* imidazole). The cells were disrupted using a Low Temperature Ultra-High-Pressure Cell Crusher (JNBIO) at 160 MPa. The cell-lysate supernatant was loaded onto a 5 ml HisTrap Excel column (GE Healthcare) mounted on an FPLC (AKTApurifier, GE Healthcare). The column was washed with five column volumes of binding buffer and eluted with elution buffer (20 m*M* Tris base pH 8.0, 500 m*M* NaCl, 500 m*M* imidazole). The recombinant protein was used for protein crystallization and further functional assays.

### 2.2. Gel-filtration standard analysis of recombinant IMP

To identify whether the purified recombinant IMP was present as a monomer or a multimer in the soluble form, the Gel Filtration Calibration Kit LMW (Cytiva) was used to determine the molecular weight of IMP. The elution value (*V*_o_) of the size-exclusion chromatography (SEC) column (ENrich SEC 650 10 × 300/Superdex 75 10/300 GL) was measured using blue dextran 2000 with phosphate-buffered saline (PBS). Calibration curves were obtained using four proteins with molecular weights (aprotinin, ribonuclease A, carbonic anhydrase and conalbumin) in the range 6500–75 000 Da. The IMP sample was finally applied onto the SEC column to measure its elution value.

### 2.3. Crystallization, data collection and structure determination of IMP

For crystallization, purified IMP was dialyzed against crystallization buffer (20 m*M* Tris base pH 8.0, 100 m*M* NaCl) and concentrated to 60 mg ml^−1^. Crystals of IMP were obtained by the sitting-drop vapour-diffusion method with buffer consisting of 0.2 *M* sodium acetate, 0.1 *M* sodium cacodylate pH 6.5, 30% PEG 8000 at 20°C. Using ethylene glycol (final concentration 15%) as a cryoprotectant, native X-ray diffraction data were collected from IMP crystals on BL-15A at NSRRC, Taiwan and processed using *HKL*-2000 (Otwinowski & Minor, 1997 ▸).

Since IMP shows little sequence similarity to proteins of known structure, its crystal structure could not be determined directly using molecular replacement (MR). IMP lacks methionine residues and does not have a cysteine or histidine residue that is capable of heavy-atom binding. Although a diffraction data set was collected to 2.0 Å resolution from a native crystal, the phase angles remained to be calculated. Attempts to incorporate methionine and cysteine residues by site-specific mutagenesis for the preparation of heavy-atom derivatives were not successful, and neither were efforts to express and crystallize protein homologues from other species. Instead, thanks to the recent development and availability of *AlphaFold*2 (Jumper *et al.*, 2021 ▸), the structure was eventually solved by MR using a theoretical model predicted using this software. The diffraction and refinement statistics of the IMP crystal are shown in Table 1 ▸. *UCSF Chimera* (https://www.cgl.ucsf.edu/chimera/) was used for structural analyses and figure generation. The structure of IMP was deposited in the Protein Data Bank (PDB) as entry 8j8y.

### 2.4. Plant materials and growth conditions

*Arabidopsis thaliana* Columbia ecotype (Col-0) and GFP-PHYL1 plants (line NF) were used in this study. GFP-PHYL1 plants were generated as described by Yang *et al.* (2015 ▸). The *Arabidopsis* seeds were surface-sterilized and vernalized at 4°C for two days before sowing on Murashige and Skoog (MS) medium with or without antibiotics for selection. One-week-old seedlings were transferred to soil and grown under long-day photoperiods (16 h light/8 h dark) at 20–25°C in a growth chamber. For inoculation with peanut witches’ broom (PnWB)-causing phytoplasm, two-month-old healthy *Catharanthus roseus* plants were grafted with a PnWB-infected branch (Liu *et al.*, 2014 ▸, 2015 ▸). Tissue from 5–6 weeks post-grafting PnWB-infected *C. roseus* were used for *in vivo* immunoprecipitation (IP).

### 2.5. Western blot

The plant extracts or recombinant proteins were denatured in two volumes of 2× sample buffer (2% SDS, 10% glycerol, 1% β-mercaptoethanol, 0.05% bromophenol blue, 50 m*M* Tris–HCl pH 6.8) and boiled at 100°C for 10 min. The proteins were separated by electrophoresis on an SDS–polyacrylamide gel and the samples were then transferred to a PVDF membrane (GE Healthcare) with transfer buffer (50 m*M* Tris base, 40 m*M* glycine, 1 m*M* SDS, 20% methanol). The IMP antibodies which were generated in this study were used as the first primary antibodies, and an HRP-conjugated secondary antibody (GE Healthcare) was used at 1:10 000 dilution. Immunostained proteins were detected using the WesternBright ECL kit (Advansta). To monitor the infection process from flower to leafy flower to leaf, the SDS–PAGE gel image with Coomassie Blue staining of Rubisco was used as a loading control for leafy flower formation. Rubisco is a key enzyme in photosynthesis that is enriched in plant leaf. In plant biology, staining for Rubisco has been recognized as an appropriate loading control due to its high abundance in cells with chloro­plasts (Zess & Kamoun, 2019 ▸).

### 2.6. *In vivo* co-immunoprecipitation assay

For the *in vivo* co-immunoprecipitation assay, 1 g of fresh healthy and PnWB-infected *C. roseus* flowers was collected and ground in liquid nitrogen. 1 ml co-precipitation buffer (25 m*M* Tris–HCl pH 7.5, 150 m*M* NaCl, 1 m*M* EDTA, 5% glycerol), 50 µl protein A agarose beads (Santa Cruz Biotechnology) and 1 µl anti-bait antibody were then added to each sample and the reaction was incubated on a rotator for 1 h at 4°C. After incubation, the reaction was centrifuged at 300*g* for 1 min, the supernatant was removed and the immunoprecipitate was washed with wash buffer (25 m*M* Tris–HCl pH 7.5, 150 m*M* NaCl, 1 m*M* EDTA, 5% glycerol, 0.1% Triton X-100) three times. The immunoprecipitate was reserved and two volumes of 2× sample buffer (2% SDS, 10% glycerol, 1% β-mercaptoethanol, 0.05% bromophenol blue, 50 m*M* Tris–HCl pH 6.8) were added for Western blot analysis.

### 2.7. Cross-linking analysis of PHYL1 and IMP

For cross-linking analysis, recombinant PHYL1 and IMP were dialyzed against 1× PBS. Subsequently, IMP and PHYL1 were mixed at a 30 µ*M* concentration of each. For control reactions, IMP and PHYL1 alone were also prepared separately. After incubation for 15 min at 20°C, bis(sulfosuccinimidyl)suberate (final concentration 1 m*M*) was added to the reactions. The reactions were incubated at the same temperature for a further 60 min and analysed by SDS–PAGE. The samples were then transferred to a PVDF membrane (GE Healthcare) with transfer buffer (50 m*M* Tris base, 40 m*M* glycine, 1 m*M* SDS, 20% methanol). Anti-IMP and anti-PHYL1 antibodies were used as the primary antibodies at 1:5000 dilution, and an HRP-conjugated secondary antibody (GE Healthcare) was used at 1:5000 dilution. The immuno­stained proteins were detected using the WesternBright ECL kit (Advansta).

### 2.8. Protein–protein docking based on a hybrid algorithm of template-based modelling

The protein–protein docking process was performed using the *HDOCK* server (https://hdock.phys.hust.edu.cn/), an open online integrated suite for bioinformatic incorporation and fast macromolecular docking (Yan *et al.*, 2020 ▸). The crystal structure of IMP was defined as the receptor for the docking of IMP and PHYL1 (PDB entry 6inr) to generate the IMP–PHYL1 complex. The structure of the plant F-actin filament from *Zea mays* pollen (PDB entry 6iug) was defined as the receptor for the docking of F-actin and IMP, of F-actin and TLNRD1 (PDB entry 6xz4), of F-actin and TLNRD1_4H (PDB entry 6xz3, a conserved domain of the TLNRD1 four-helix bundle; amino acids 148–270) and of F-actin and the IMP–PHYL1 complex. The *HDOCK* server empirically defined the confidence score using a knowledge-based iterative scoring function ITScorePP or ITScorePR. The likeliness of two molecules binding is indicated by the equation

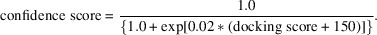




Generally, when the confidence score is larger than 0.7 the server has high confidence that the predicted model of the receptor and ligand will bind, and when the confidence score is between 0.5 and 0.7 the server suggests that the input receptor and ligand will bind. However, the confidence score is still only suggested to serve as a relevant reference due to its empirical nature.

## 3. Results

### 3.1. Structural information on IMP provides further evidence for the interaction of IMP and F-actin

**Figure 1:**
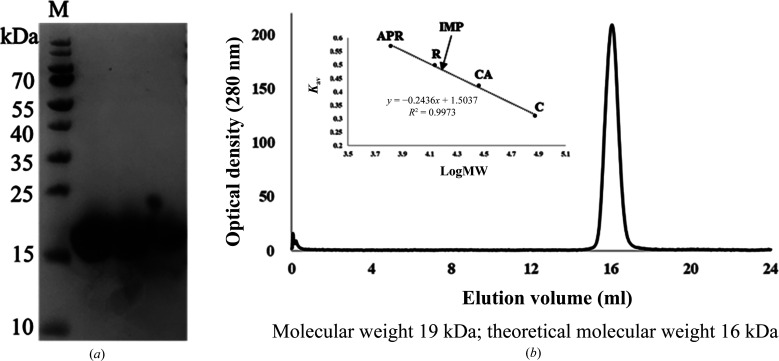
Purification and gel-filtration analysis of recombinant IMP. (*a*) SDS–PAGE of purified recombinant IMP on a 12% SDS–PAGE gel. The samples were loaded with 12 µl in each well. Lane M contains molecular-weight markers (labelled in kDa). (*b*) Gel-filtration standards for recombinant IMP. The calibration standard curve for size-exclusion chromatography was measured using the standard substances aprotinin (APR, 6.5 kDa), ribonuclease A (R, 13.7 kDa), carbonic anhydrase (CA, 29 kDa) and conalbumin (C, 75 kDa).

Previous results indicated that IMP and F-actin can interact with each other and that this interaction is biologically significant. To provide further evidence for IMP–actin interaction, we sought to determine the crystal structure of IMP without the N-terminal transmembrane domain. High-purity recombinant IMP protein with a molecular weight of ∼19 kDa was expressed and purified. As shown in Fig. 1 ▸, to clarify whether two IMP monomers have the ability to form a dimer, we performed gel-filtration analysis to measure the native molecular weight of IMP. Gel-filtration analysis gave a calculated molecular weight for IMP (∼19/∼22 kDa) close to the theoretical molecular weight (∼16 kDa), indicating that IMP is present as a monomer [Fig. 1 ▸(*b*) and Supplementary Fig. S1]. We therefore used the IMP monomer in subsequent structural analysis.

**Figure 2:**
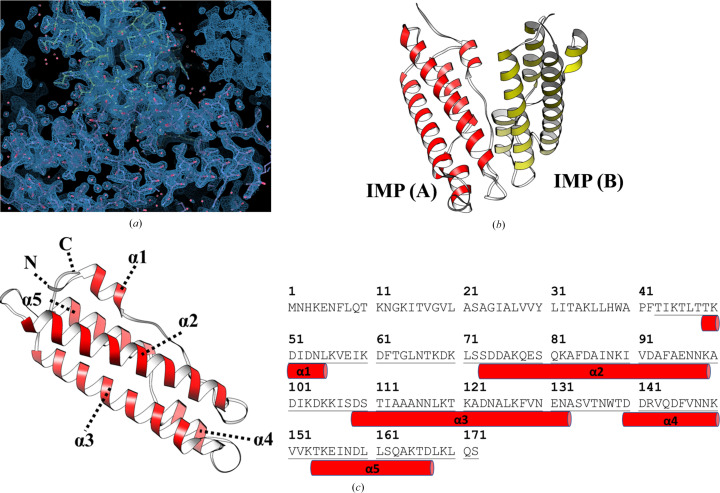
The crystal structure of recombinant IMP. (*a*) Refined electron-density map of the IMP crystal. (*b*) Ribbon diagram of the IMP dimer in the unit cell. (*c*) The secondary-structural elements of the IMP monomer. The red cylinders representing α-helices are labelled in parallel with the sequence.

Diffraction data were collected from a native IMP crystal to 2.0 Å resolution and the structure was solved by molecular replacement using an *AlphaFold*2 model, producing clear electron-density maps [Fig. 2 ▸(*a*)]. The hexagonal crystal of IMP belonged to space group *P*6_5_ and each asymmetric unit contained two monomers [Table 1 ▸, Fig. 2 ▸(*b*)]. Each IMP monomer contains five α-helices that fold into an antiparallel α-helix bundle [Fig. 2 ▸(*c*)]. The N-terminal helix is short and the C-terminal helix is divided into two segments, making the bundle four-membered at one end and three-membered at the other. Furthermore, when the IMP structures from *AlphaFold* prediction and X-ray analysis were compared, the r.m.s.d. between 125 pruned C_α_ atom pairs is 0.49 Å (0.65 Å across all 129 pairs), indicating high similarity. Only the side chains in the N- and C-terminal regions show some limited variations, indicating the reliability and accuracy of *AlphaFold* as a useful tool to propose the structures of phytoplasma proteins (Supplementary Fig. S2).

The IMP monomers are almost identical in structure, with an r.m.s.d. of only 0.27 Å between 126 pairs of C_α_ atoms. The structure is also stabilized by the formation of an extensive hydrophobic core inside the helical bundle, which is contributed by nonpolar side chains (Leu47, Ile52, Leu55, Val57, Ile59, Phe62, Leu71, Ala76, Ala83, Ala86, Ile87, Ile90, Val91, Phe94, Ile107, Ile112, Ala115, Leu118, Ala122, Ala125, Leu126, Phe128, Val129, Trp1138, Val143, Phe146, Val147, Val151, Val152, Ile157, Leu160, Leu161, Ala164, Leu165 and Leu170). The involvement of both N-terminal and C-terminal segments in the hydrophobic core suggests considerable resistance to unfolding. The surface areas of the two monomers are 7480 and 7257 Å^2^, but the interface buries only 333 Å^2^ (4%) of the molecular surface and involves no more than a handful of polar residues from each monomer. The second largest crystal contact has an interface area of 288 Å^2^ and the third largest crystal contact has an interface of only 149 Å^2^. Apparently, IMP itself does not form a dimer or a higher oligomer, which is consistent with biochemical observations that this protein may function as a monomer.

**Figure 3:**
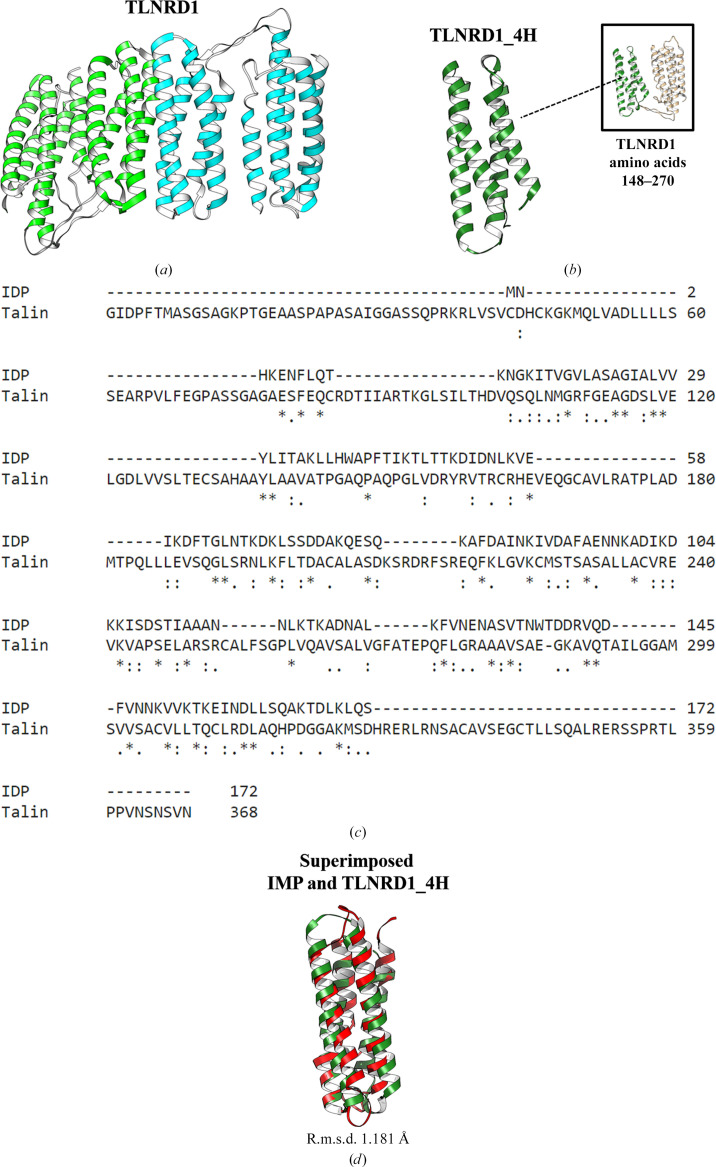
Structural analysis of the IMP–PHYL1 model and homologue comparison of the IMP structure. (*a*) The dimeric structure of TLNRD1; the two monomers of the rod domain are presented as green and cyan ribbons. (*b*) The structure of the TLNRD1_4H bundle (dark green ribbon; amino acids 148–270). (*c*) Alignment of the IMP and TLNRD1 protein sequences, which show relatively low sequence identity. (*d*) The models of IMP and TLNRD1_4H superimposed with an r.m.s.d. of 1.181 Å.

For the identification of a potent functional homologue to IMP, we next performed a structural homology search for the IMP monomer using the *DALI* server (Holm, 2022 ▸). The results show that IMP shares similarity with talin rod domain-containing protein 1 (TLNRD1) [Fig. 3 ▸(*a*); PDB entry 6xz4, *Z*-score 9.9, r.m.s.d. 3.1 Å]. Unlike in IMP, two TLNRD1 monomers assemble into a dimer. Each TLNRD1 monomer contain two copies of an α-helix bundle [TLNRD1_4H bundle; Fig. 3 ▸(*b*)], which is similar to IMP. Structural alignment of IMP and TLNRD1_4H shows an r.m.s.d. of 1.181 Å between 32 pruned atom pairs [Fig. 3 ▸(*d*)] (6.044 Å across all 108 pairs) using *MatchMaker* from *UCSF Chimera* (Meng *et al.*, 2006 ▸).

**Figure 4:**
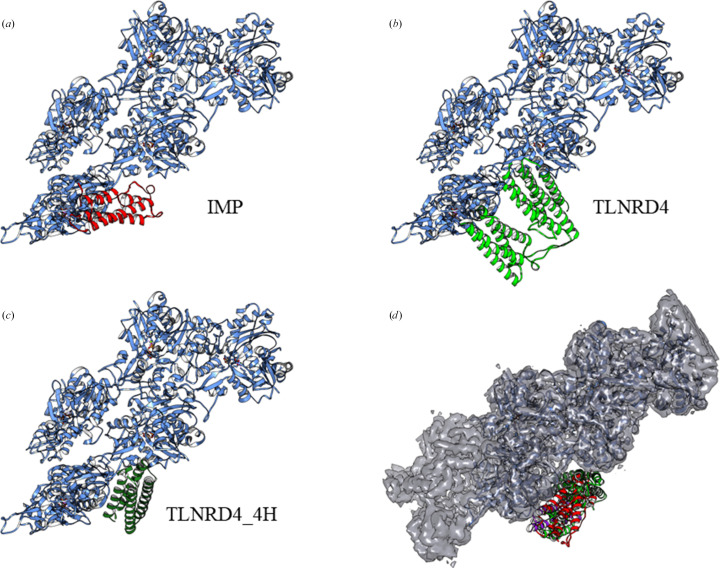
Calculated structural modelling of potent F-actin-binding proteins. (*a*) The proposed IMP–actin binding model from the *HDOCK* server with a docking score of −155.95 and a confidence score of 0.530. The F-actin model is coloured blue, while the IMP model is coloured red. (*b*) The proposed TLNRD1–actin binding model from the *HDOCK* server with a docking score of −262.13 and a confidence score of 0.904. The F-actin model is coloured blue, while the TLNRD1 model is coloured light green. (*c*) The proposed TLNRD1_4H–actin binding model from the *HDOCK* server with a docking score of −260.80 and a confidence score of 0.902. The F-actin model is coloured blue, while the TLNRD1_4H model is coloured dark green. (*d*) The alignment of all docking ligands (IMP, TLNRD1, TLNRD1_4H and IMP/PHYL1) to F-actin (solid ribbons) with the electron-density maps from the PDB structures.

In a previous study, TLNRD1 was confirmed to be an F-actin-binding protein (Cowell *et al.*, 2021 ▸). Since IMP also has the ability to bind actin, these two proteins are functionally similar in addition to their structural similarity (Petrey *et al.*, 2009 ▸). The resulting binding model of IMP and F-actin has a reasonable structural complementarity at the interaction interface [Fig. 4 ▸(*a*)], with a confidence score of 0.530; thus, from the docking result the server infers that IMP and plant F-actin could possibly form a complex. Here, Lys113 and Asp117 of IMP form hydrogen bonds to Gln356 of actin, Ser120 of IMP forms hydrogen bonds to Gln355 of actin and Thr124 of IMP forms hydrogen bonds to Glu366 of actin. These residues appear to constitute a stable interface for the binding of IMP and actin. To further evaluate the stability of the IMP–actin complex, the structures of TLNRD1 and TLNRD1_4H were further analysed using the *HDOCK* server in order to model the TLNRD1–actin structure. The result showed that TLNRD1 has a large interface with F-actin with a confidence score of 0.904, indicating that these two molecules have a high potential to bind to each other [Fig. 4 ▸(*b*)]. Similar results were also obtained from the docking model of TLNRD1_4H with F-actin, with a high confidence score of 0.902 [Fig. 4 ▸(*c*)]. Generally, all of the top *HDOCK* solutions for IMP, TLNRD1 and TLNRD1_4H docked with F-actin from *Z. mays* pollen show a concerted binding model in the groove of the actin helix (Supplementary Fig. S3). One of the limitations of this docking method is caused by the receptor composition. F-actin is a long-chain helical polymer that concludes with repeated G-actins, but the receptor structure (PDB entry 6iug) only contains five single actin molecules. As a result, the two end positions of the actin become ideal low-free-energy binding positions. Accordingly, docking modes with the ligand located at the two ends of the receptor F-actin, such as IMP rank 1/2/3/4/9 and TLNRD1 rank 5, should be excluded. However, all of the other top-ten ranking models show consistency in the confidence score given by the *HDOCK* server. For IMP and TLNRD1_4H all models scored 0.6–0.7, while the TLNRD1 models scored 0.8–0.9. The highly consistent binding modes of IMP and TLNRD1_4H provide reliable results indicating that the input ligands would interact with the F-actin structure.

### 3.2. IMP was identified as a potential PHYL1-interacting protein (PIP)

Recently, our parallel study of potential interaction candidates for the phytoplasma effector PHYL1 also revealed the importance of IMP. PHYL1 has been confirmed as the key factor in phytoplasma-induced flower-like leaves. By interacting with different proteins, PHYL1 affects the control mechanisms of plant flowering. For instance, PHYL1 interacts with MADS transcription factors (MTFs) related to flowering such as SEP3 (SEPALLATA3) and AP1 (MacLean *et al.*, 2014 ▸; Maejima, Iwai *et al.*, 2014 ▸; Maejima *et al.*, 2015 ▸). Furthermore, by interacting with RAD23, PHYL1 mediates MTF-ubiquitin­ated degradation, resulting in leafy flower formation (MacLean *et al.*, 2014 ▸; Maejima, Iwai *et al.*, 2014 ▸). Our previous study also revealed the interaction mode of PHYL1 and MTFs using proteomic and structural approaches (Liao *et al.*, 2019 ▸).

In subsequent studies of PHYL1, we attempted to utilize *in vivo* PHYL1 immunoprecipitation with LC-MS/MS (*n* = 3) to explore whether PHYL1 has the ability to further interact with other proteins. The result was the identification of 103 potential PHYL1-interacting proteins (PIPs) from PnWB-infected *C. roseus* plants (Supplementary Table S1). Interestingly, IMP and its binding partners, actin and tubulin, were also found to be PIPs. It is not surprising that most of the PIPs were *C. roseus* proteins, such as the gibberellin-regulated GASA/GAST/Snakin family protein, d-ribulose-5-phosphate-3-epimerase and the cytoskeleton protein F-actin 7 (Supplementary Table S1). However, the detection of the abundant membrane protein IMP as a PIP attracted our attention. Prior to this study, interaction between PHYL1 and IMP had not been confirmed. We thus suspect that the PHYL1–IMP interaction may have a functional role related to F-actin in phytoplasma pathogenesis.

**Figure 5:**
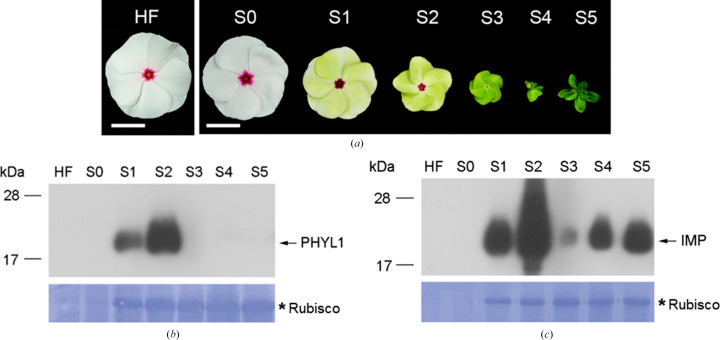
Detection of the expression of PHYL1 and IMP in PnWB-infected *C. roseus*. (*a*) The various leafy flower symptoms of PnWB-infected *C. roseus*. (*b*, *c*) Expression levels of PHYL1 and IMP at various stages of leafy flower symptoms. Asterisks indicate Rubisco staining as a comparable quality loading control for leafy flower formation.

In order to confirm the role of IMP in the phytoplasma-infection process, we first evaluated the expression levels of IMP at different stages (S1–S5) of PnWB-infected *C. roseus* flowers. The leafy flower symptoms of PnWB-infected *C. roseus* were confirmed by grafting two-month-old healthy *C. roseus* plants onto a PnWB-infected branch [Fig. 5 ▸(*a*)]. Next, an *in vivo* Western blot with anti-PHYL1 or anti-IMP from the tissue of PnWB-infected *C. roseus* was performed to examine the expression levels of PHYL1 and IMP [Figs. 5 ▸(*b*) and 5 ▸(*c*)]. Rubisco staining shows leafy flower formation from S1 to S5, indicating the process of PnWB infection. IMP showed clear signals from S1 to S5 but was abundant in S2. These data indicate that IMP is expressed in the same phase transitions during PnWB infection.

**Figure 6:**
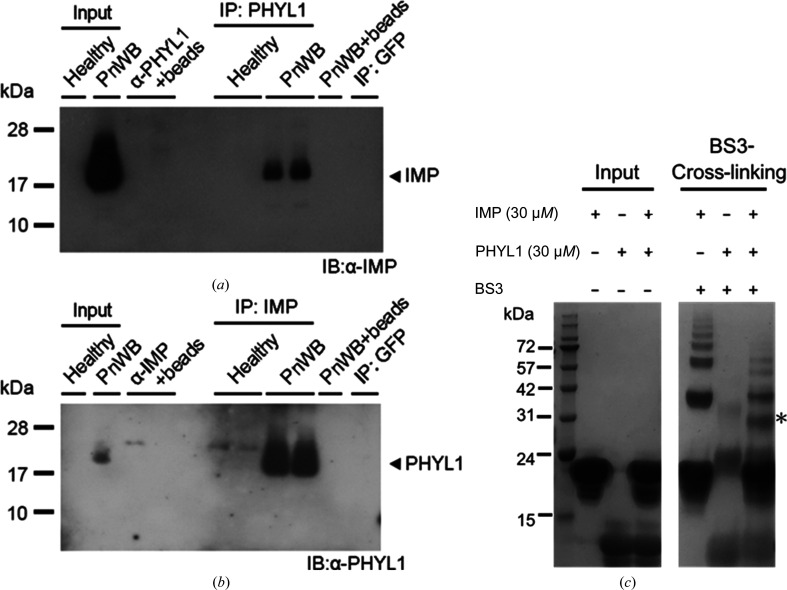
Confirmation of the PHYL1–IMP interaction. (*a*, *b*) The *in vivo* co-immunoprecipitation of PHYL1 (*a*) and IMP (*b*) from PnWB-infected *C. roseus* plants. α-PHYL1 was diluted 1:5000 for detection. α-IMP was diluted 1:40 000 for detection. (*c*) Cross-linking analysis of PHYL1 and IMP. The shifting band due to protein interactions is marked with an asterisk.

Next, an *in vivo* IP with anti-PHYL1 antibody from the PnWB-infected *C. roseus* tissue showed that PHYL1 could interact with IMP [Fig. 6 ▸(*a*)]. Consistently, IP of IMP from the PnWB-infected samples detected PHYL1 [Fig. 6 ▸(*b*)]. An *in vitro* cross-linking assay was also used to further confirm the PHYL1–IMP interaction [Fig. 6 ▸(*c*) and Supplementary Fig. S4]. The cross-linker bis(sulfosuccinimidyl)suberate (BS3) was used in this assay. BS3 connects amine groups on lysine residues with a distance less than its arm length (11.4 Å). If IMP has the ability to interact with PHYL1, their lysine residues are likely to come close to each other and be cross-linked by BS3. As shown in Fig. 6 ▸(*c*), the cross-linked IMP and PHYL1 were shifted upwards and the major shifted protein band was around 31 kDa (indicated by an asterisk). The molecular weight of this shifted band is close to the sum of those of IMP (∼16 kDa) and PHYL1 (∼14 kDa). In the subsequent Western blot experiment (Supplementary Fig. S4), the shifted band can be recognized by an anti-IMP antibody, which produces a strong signal. The same band was also recognized by an anti-PHYL1 antibody with a mild signal. We suspect that the cross-linking may affect recognition by the PHYL1 antibody. Nevertheless, because the band shift was obvious and can only be seen in the lane containing a PHYL1/IMP mixture, we believe that the shifted band was caused by a 1:1 binding stoichiometry of the two proteins.

### 3.3. Structural analysis revealed that IMP–PHYL1 can form a complex with F-actin

For clarification of the IMP–actin and IMP–PHYL1 interactions, we first proposed a binding model for IMP–PHYL1 using the *HDOCK* server. The result shows that IMP and PHYL1 have a reasonable structural complementarity at the interaction interface, with a high confidence score of 0.729 [Supplementary Fig. S5(*a*)], suggesting that IMP has the ability to bind to PHYL1. We next used IMP–PHYL1 as a ligand model for docking with F-actin using the *HDOCK* server. The result showed a similar docking model with a confidence score of 0.726 [Supplementary Fig. S5(*b*)].

It is noteworthy that all of the docking results of the tested proteins in this study (IMP, TLNRD1, TLNRD1_4H and IMP–PHYL1) show consistent binding models. All of the interfaces between the ligands and the defined receptor (*Z. mays* pollen F-actin filaments) are located in the same groove of the F-actin polymer. Judging by these *in silico* analysis results, in conjunction with the previous *in vitro*/*in vivo* experimental data, we are confident about the binding affinity of IMP to F-actin filaments.

## 4. Discussion

**Figure 7:**
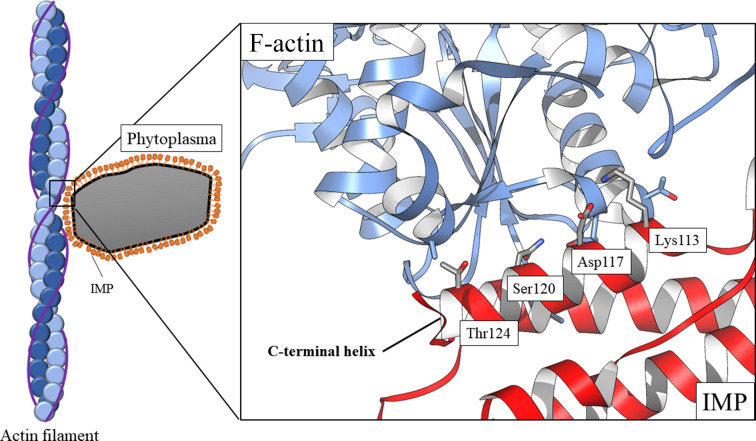
Schematic model of phytoplasma binding to plant F-actin.

In this study, we provide new insights into the molecular mechanism of phytoplasma immunodominant membrane protein (IMP). In previous studies, when IMP was expressed in plants the transgenic plants did not show relevant symptoms after phytoplasma infection (Boonrod *et al.*, 2012 ▸; Konnerth *et al.*, 2016 ▸). Another study also indicated that IMP promotes the transmission of wheat blue dwarf (WBD) phytoplasma by directly interacting with α-tubulin in leafhoppers (Ding *et al.*, 2022 ▸). These results suggest that IMP is important for the survival or movement of phytoplasma by binding to cyto­skeleton proteins. Here, we present the first crystal structure of IMP (Fig. 2 ▸). Interestingly, although alignment of their sequences shows low sequence identity between IMP and TLNRD1 [Fig. 3 ▸(*c*)], a homology search using the *DALI* server reveals high structural similarity of the two proteins [Fig. 3 ▸(*d*)]. The structural similarity between IMP and TLNRD1 provides a basis for their common inter­action with F-actin. Alignment of the docking results [Fig. 4 ▸(*d*)] shows that IMP and TLNRD1 have similar binding modes in the groove region of the actin filaments. From our docking models (Fig. 4 ▸), we inferred that IMP might function in intercellular F-actin attachment and as a structural anchor for phytoplasmas in plant cells (Fig. 7 ▸). In a recent study, a cluster of amino acids in the C-terminus of IMP were suggested to be an actin-binding region (Boonrod *et al.*, 2023 ▸). This result is consistent with our IMP–actin docking model in that IMP utilized its C-terminal Lys113, Asp117, Ser120 and Thr124 to interact with the F-actin filament (Fig. 7 ▸).

Additionally, we found that IMP could also interact with PHYL1. Through proteomic studies to identify potential PHYL1-interacting proteins (PIPs; Supplementary Table S1), we revealed several cellular mechanisms that may be affected by PHYL1. For instance, phytoplasmas highly depend on metabolic compounds from the host and lack amino-acid biosynthesis genes (Oshima *et al.*, 2013 ▸; Maejima, Oshima *et al.*, 2014 ▸). Two potential PHYL1-interacting proteins, GLN2 and GLDP1, are involved in amino-acid biosynthesis. In addition, high levels of reactive oxygen species have been detected in phytoplasma-infected mulberry plants as part of the defence response (Takemoto *et al.*, 2006 ▸; Ji *et al.*, 2009 ▸, Miura *et al.*, 2012 ▸). Potential PHYL1–APX4 or PHYL1–CSD2 interactions can be used in scavenging reactive oxygen species (Xing *et al.*, 2013 ▸; Wang *et al.*, 2014 ▸). All of these proteins might be suitable candidates for studying phytoplasma infection. Notably, IMP and its binding partners, actin and tubulin, were also found to be PIPs. We subsequently confirmed the IMP–PHYL1 interaction by *in vivo* immunoprecipitation and an *in vitro* cross-linking assay. Nevertheless, we have to admit that IMP and PHYL1 show relatively weak binding in other *in vitro* methods (for example gel-filtration analysis; data not shown). We are considering two possible reasons that might explain this situation. Firstly, under *in vivo* conditions there may be some other effectors that enhance the binding of IMP and PHYL1. Secondly, these two proteins might have only a transient interaction. Therefore, it is difficult to detect a stable IMP–PHYL1 complex or a clear peak shift in the gel-filtration analysis. Given that several key biological events have been identified to adopt a ‘hit-and-run’ strategy between two binding partners based on their transient interaction (Chichili *et al.*, 2013 ▸), the interaction between IMP and PHYL1 might also follow this pattern.

In order to provide further evidence for this interaction, we performed a docking analysis (Supplementary Fig. S5). We notice that IMP and PHYL1 share an antiparallel helices arrangement, and in our docking analysis they assemble into a larger α-helical bundle (Supplementary Fig. S5*a*). Interestingly, the IMP–PHYL1 (confidence score 0.726) model showed consistency with that of IMP alone (confidence score 0.530) as well as with TLNRD1 (confidence score 0.940) in the F-actin-binding model (Supplementary Fig. S5*b*). Compared with the IMP alone model, the IMP–PHYL1 model had an apparently increased confidence score for the docking result. It is suggested that PHYL1 may also be involved in regulation of the interaction of IMP and actin. We suggest that IMP might function in intercellular F-actin attachment and as a structural anchor for phytoplasmas in plant cells. Meanwhile, IMP might serve as an accumulation point to recruit other pathogenic effectors such as PHYL1. In conclusion, the results of this study may help us to further clarify the function of IMP and provide new insights into the pathological mechanism of phytoplasmas.

## Supplementary Material

PDB reference: phytoplasma immunodominant membrane protein, 8j8y

Supplementary Figures and Tables. DOI: 10.1107/S2052252524003075/lz5068sup1.pdf

## Figures and Tables

**Table 1:** Data-collection and refinement statistics for IMP Values in parentheses are for the outer shell.

Data collection	
Wavelength (Å)	1.000
Space group	*P*6_5_
*a*, *b*, *c* (Å)	96.5, 96.5, 54.2
α, β, γ (°)	90, 90, 120
Resolution (Å)	50–2.00 (2.07–2.00)
Unique reflections	19496 (1922)
Multiplicity	4.6 (3.7)
Completeness (%)	99.8 (99.6)
〈*I*/σ(*I*)〉	26.3 (2.5)
CC_1/2_	0.952 (0.780)
*R*_merge_ (%)	5.8 (53.2)
Refinement	
*R*_work_ (%)	18.0
*R*_free_ (%)	22.2
R.m.s.d., bond lengths (Å)	0.0025
R.m.s.d., angles (°)	0.55
No. of atoms	2404
Mean *B* factor (Å^2^)	37.3
Ramachandran plot	
Most favoured (%)	98.4
Allowed (%)	1.6
Outliers (%)	0
