# Supplementary material for: Structural insights into the molecular mechanism of phytoplasma immunodominant membrane protein
Source: IUCrJ. 2024 Apr 24;11(Pt 3):384–94. doi: 10.1107/S2052252524003075 (PMC11067747; doi:10.1107/S2052252524003075)
Supplement: Supplementary file 1 [file m-11-00384-sup1.pdf]

IUCrJ

Volume 11 (2024)

Supporting information for article:

Structural insights into the molecular mechanism of phytoplasma immunodominant membrane protein

Chang-Yi Liu, Han-Pin Cheng, Chan-Pin Lin, Yi-Ting Liao, Tzu-Ping Ko, Shin-Jen Lin, Shih-Shun Lin and Hao-Ching Wang

Table S1 The list for potential PHYL1-interacting proteins.

DDS ID	AGI	Protein name	Abbreviation	PubMed ID
**Photosynthesis**				
DDS57960	AT2G39730.1	Rubisco activase	RCA	25849391
DDS57462	ATCG00490.1	Ribulose-bisphosphate carboxylases	RBCL	26025532
DDS41686	ATCG00120.1	ATP synthase subunit alpha	ATPA	19697039
DDS44062	AT1G56190.1	Phosphoglycerate kinase family protein	cPGK2	24930633
DDS43812	ATMG01190.1	ATP synthase subunit 1	ATP1	25835989
DDS18960	AT1G12900.1	Glyceraldehyde 3-phosphate dehydrogenase A subunit 2	GAPA-2	17854848
DDS12824	AT5G14780.1	Formate dehydrogenase	FDH	28988354
DDS19554	AT5G13120.1	Cyclophilin 20-2	CYP20-2	22041789
DDS19944	AT5G66190.1	Ferredoxin-NADP(+)-oxidoreductase 1	FNR1	22300243
DDS59400	AT5G38410.3	Rubisco small subunit 3b	RBCS3B	22223809
DDS57901	AT1G06680.1	Photosystem II subunit P-1	PSBP-1	17604269
**Defense response**				
DDS18837	AT3G12500.1	Basic chitinase	B-CHI	DDS18837
DDS14248	AT1G20510.1	OPC-8:0 CoA ligase1	OPCL1	DDS14248
DDS23169	AT1G70840.1	MLP-like protein 31	MLP31	DDS23169
DDS58113	AT3G14420.1	Glycolate oxidase 1	GOX1	DDS58113
DDS20437	AT4G37990.1	Elicitor-activated gene 3	ELI3.1	DDS20437
DDS45044	AT4G37990.1	Elicitor-activated gene 3	ELI3.2	DDS45044
DDS57458	AT2G38870.1	PR-6 proteinase inhibitor family	PR-6 PI	DDS57458
DDS30631	AT1G70840.1	MLP-like protein 31	MLP31	DDS30631
DDS11033	AT2G23620.1	Methyl esterase 1	MES1	DDS11033
DDS47142	AT5G61410.1	D-ribulose-5-phosphate-3-epimerase	RPE	DDS47142
DDS59062	AT4G37980.1	Cinnamaldehyde and hexenal reductase	CHR	DDS59062
DDS58088	AT1G74020.1	Strictosidine synthase	STR	DDS58088
**Cytoskeleton**				
DDS53318	AT5G09810.1	Actin 7	ACT7	29531328
DDS19330	AT5G19780.1	Tubulin alpha-5	TUA5	1498608
DDS52148	AT1G50010.1	Tubulin alpha-2 chain	TUA2	30833711
DDS18929	AT4G38510.5	V-ATPase B subunit 2	VAB2	22371505
**Cellular respiration**				
DDS48404	AT1G59900.1	Pyruvate dehydrogenase complex E1 alpha	E1 alpha	7590338
DDS57166	AT2G36530.1	Enolase	ENO2	25620024
DDS50893	AT3G13930.1	Mitochondrial pyruvate dehydrogenase subunit 2-2	MTE2-2	26194327
DDS11268	AT2G44350.2	Citrate synthase 4	CS4	22115780
DDS52367	AT1G42970.1	Glyceraldehyde-3-phosphate dehydrogenase B subunit	GAPB	11442054

**Intracellular protein transport**				
DDS19123	AT4G02080.1	Secretion-associated RAS super family 2	SAR2	12242382
DDS51812	AT5G55190.1	Ran GTPase 3	RAN3	17530257
DDS19827	AT1G45000.1	AAA-type ATPase family protein	AAA-type ATPase	20118269
**Cell division**				
DDS51921	AT5G20720.1	Chaperonin 20	CPN20	21633907
DDS13025	AT5G03340.1	Cell division cycle 48C	CDC48C	12427991
**Amino acid metabolism**				
DDS11601	AT3G19480.1	Phosphoglycerate dehydrogenase 3	PGDH3	24058165
DDS11169	AT5G35630.1	Glutamine synthetase 2	GLN2	30368031
DDS44040	AT4G33010.1	Glycine decarboxylase P-protein 1	GLDP1	26603271
DDS19141	AT4G13930.1	Serine hydroxymethyltransferase 4	SHM4	16951055
DDS53279	AT4G14880.1	O-acetylserine (thiol) lyase isoform A1	OAS-TLA1	20429919
**Fatty acid oxidation**				
DDS19389	AT2G33150.1	Peroxisomal 3-ketoacyl-CoA thiolase 3	PKT3	20463027
**ROS-scavenging**				
DDS21519	AT4G09010.1	Ascorbate peroxidase 4	APX4	24407512
DDS58222	AT5G06290.1	2-cysteine peroxiredoxin B	2-Cys PrxB	29128357
DDS57429	AT2G28190.1	Copper/Zinc superoxide dismutase 2	CSD2	31093688
**Growth and development**				
DDS14940	AT1G47480.1	alpha/beta-Hydrolases superfamily protein		17597079
DDS18899	AT3G04400.1	Embryo defective 2171	EMB2171	27247031
DDS12009	AT4G26300.1	Embryo defective 1027	EMB1027	21139083
DDS53303	AT2G18420.1	Gibberellin-regulated GASA/GAST/Snakin family protein	Snakin/GASA	22836500
**Proton pump**				
DDS58014	AT3G01390.1	Vacuolar membrane ATPase 10	VMA10	11004467
DDS18932	AT1G78900.1	Vacuolar ATP synthase subunit A	VHA-A	22943363
Protein quality control				
DDS13016	AT1G02560.1	Nuclear encoded CLP protease 5	NCLPP5	26085677
DDS18881	AT1G09130.3	ATP-dependent caseinolytic protease/crotonase family protein	CLP	18230142
DDS51845	AT4G24280.1	Chloroplast heat shock protein 70-1	CPHSC70-1	20193958
DDS51861	AT5G56030.2	Heat shock protein 81-2	HSP81-2	30194279
DDS51072	AT3G23990.1	Heat shock protein 60 (HSP60)	HSP60	20018591
DDS18094	AT3G05530.1	Regulatory particle triple-A ATPase 5A	RPT5A	31156663
DDS42413	AT5G58290.1	Regulatory particle triple-A ATPase 3	RPT3	20516081
**Carbohydrate metabolism**				
DDS46369	AT5G51970.1	Sorbitol dehydrogenase	SDH	25755662
DDS57950	AT3G57520.1	Seed imbibition 2.1	SIP2.1	20739305
DDS53269	AT3G57520.1	Seed imbibition 2.2	SIP2.2	20739305
**DNA repair**				

DDS52608	AT1G20340.1	DNA-damage-repair/toleration protein 112 (DRT112)	DRT112	19825610
**Phytoplasma protein**				
	AGT17678.1	immunodominant membrane protein	IMP	9611800
**Purine and pyrimidine metabolism**				
DDS59111	AT4G09320.1	Nucleoside diphosphate kinase 1	NDPK1	28586477
DDS57175	AT4G12440.2	Adenine phosphoribosyl transferase 4	APT4	12010467
**Detoxification**				
DDS19410	AT3G48000.1	Aldehyde dehydrogenase 2B4	ALDH2B4	19320993
**Lignin biosynthesis**				
DDS20250	AT4G39330.1	Cinnamyl alcohol dehydogenase 9 (CAD9)	CAD9	20829305
**Energy**				
DDS42971	AT2G35660.1	FAD/NAD(P)-binding oxidoreductase family protein		14576160
DDS51803	AT3G53620.1	Pyrophosphorylase 4	PPA4	22566496

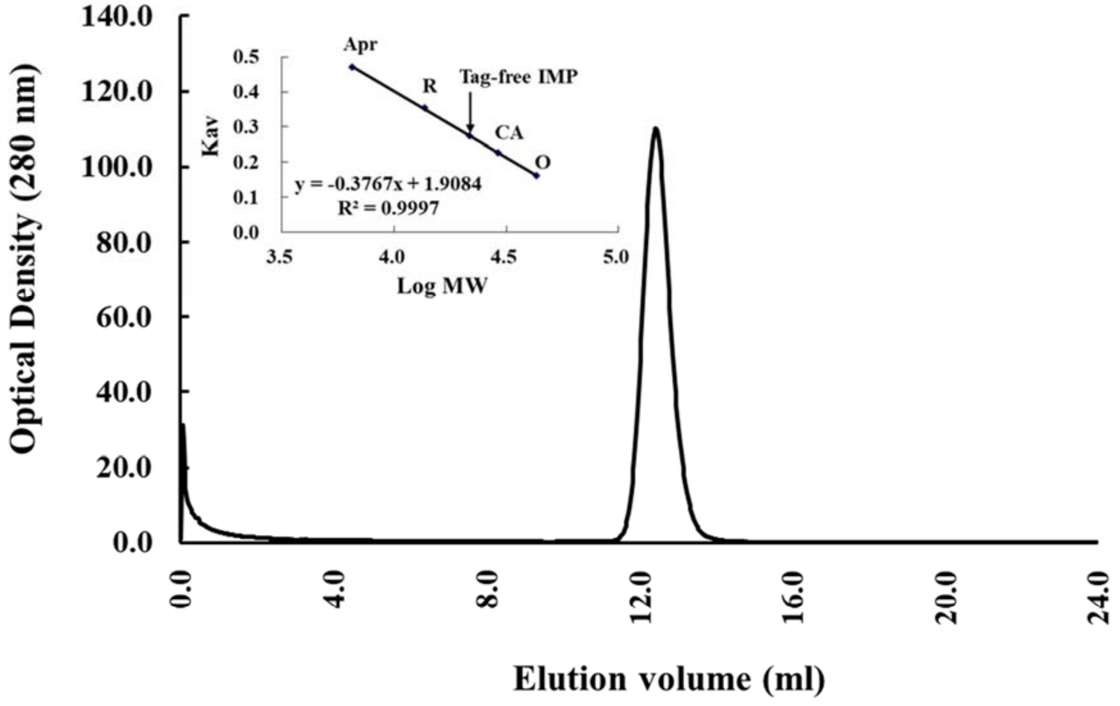

	Tag-free IMP
Theoretical MW	16.04 kDa
Calculated MW	21.66 kDa

Figure S1 Gel filtration analysis with Superdex 75 column. The standard curve for size exclusion chromatography was drawn by measuring standard proteins: Aprotinin (APR, 6.5 kDa), Ribonuclease A (R, 13.7 kDa), Carbonic anhydrase (CA, 29 kDa) and Ovalbumin (O, 43 kDa).

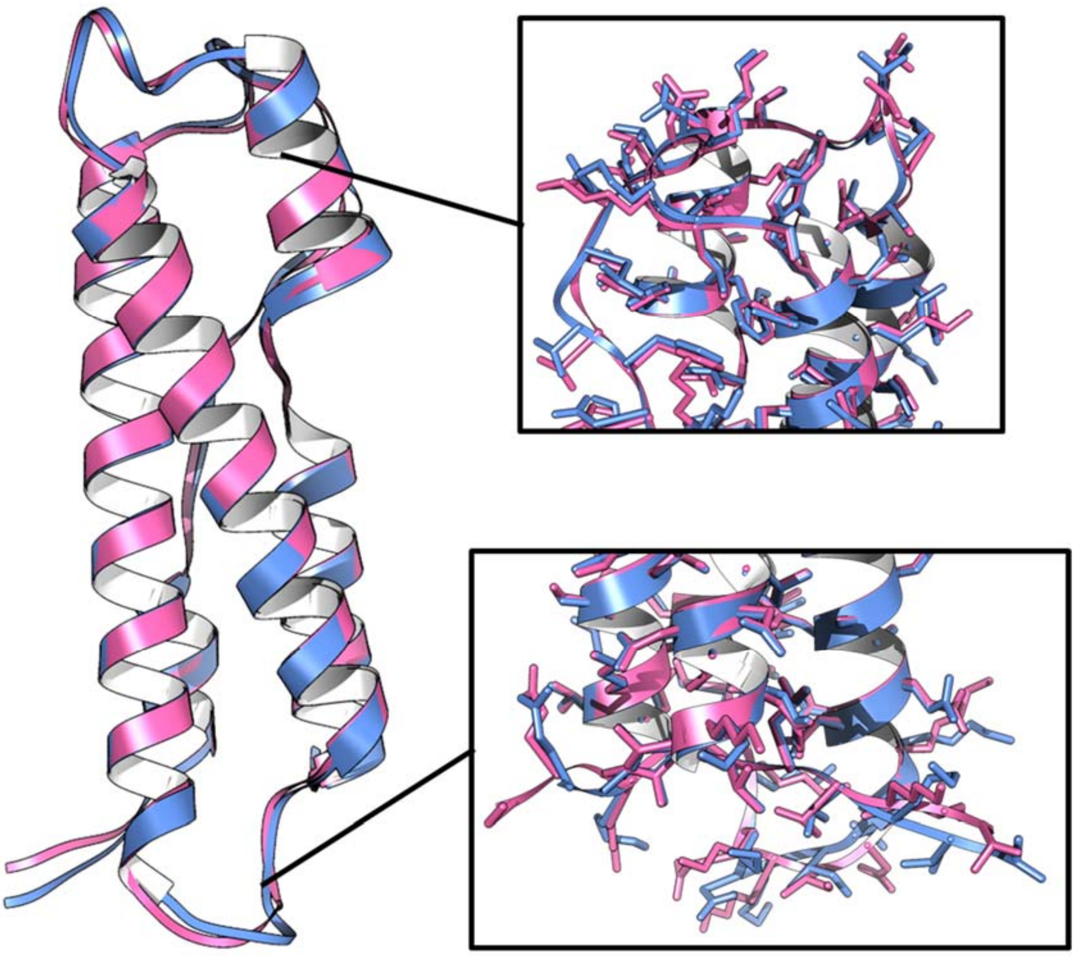

Figure S2 Comparison of IMP structures from AlphaFold prediction (pink) and X-ray analysis (light blue).

	IMP_A_with_Actin_Zea mays pollen_top10_models				
	Rank 1	Rank 2	Rank 3	Rank 4	Rank 5
	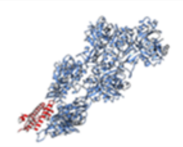	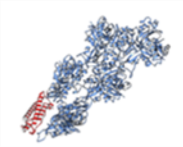	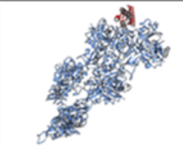	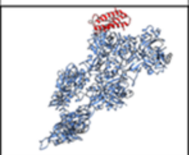	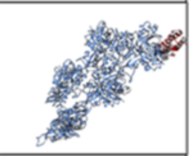
Docking Score	-186.34	-170.51	-163.56	-160.14	-159.19
Confidence Score	0.6741	0.6011	0.5674	0.5505	0.5458
Ligand rmsd (Å)	63.83	62.54	87.75	80.60	103.87
	Rank 6	Rank 7	Rank 8	Rank 9	Rank 10
	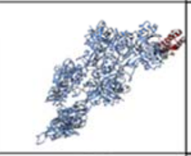	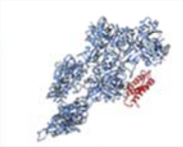	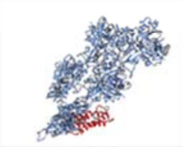	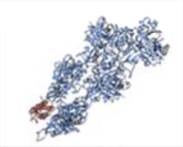	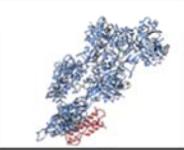
Docking Score	-157.98	-157.04	-155.95	-152.00	-151.87
Confidence Score	0.5398	0.5351	0.5297	0.5100	0.5093
Ligand rmsd (Å)	66.32	62.73	50.68	64.69	53.87

	TLNRD1_A_with_Actin_Zea mays pollen_top10_models				
	Rank 1	Rank 2	Rank 3	Rank 4	Rank 5
	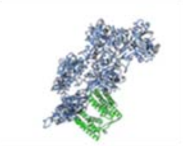	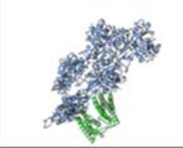	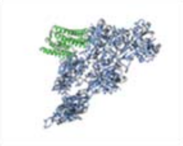	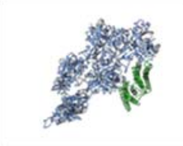	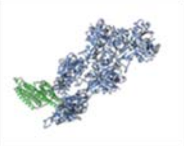
Docking Score	-262.13	-247.16	-239.56	-237.05	-235.45
Confidence Score	0.9040	0.8747	0.8571	0.8508	0.8467
Ligand rmsd (Å)	101.12	102.03	44.82	117.01	53.42
	Rank 6	Rank 7	Rank 8	Rank 9	Rank 10
	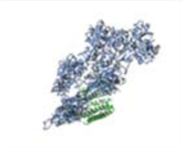	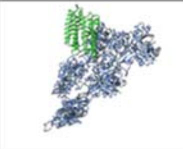	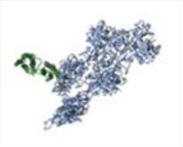	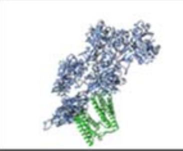	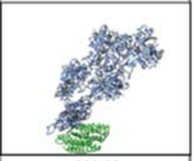
Docking Score	-233.88	-233.27	-233.07	-231.35	-230.57
Confidence Score	0.8426	0.8410	0.8404	0.8358	0.8336
Ligand rmsd (Å)	76.33	78.40	43.06	103.47	96.45

	TLNRD1_4H_A_with_Actin_Zea mays pollen_top10_models				
	Rank 1	Rank 2	Rank 3	Rank 4	Rank 5
	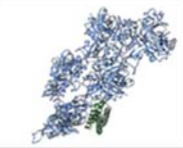	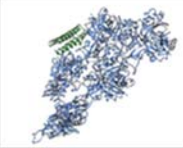	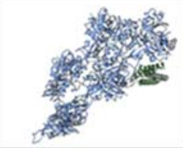	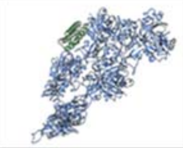	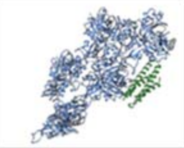
Docking Score	-168.27	-168.13	-166.98	-163.89	-163.54
Confidence Score	0.5903	0.5897	0.5841	0.5690	0.5673
Ligand rmsd (Å)	101.39	103.91	103.92	55.88	93.85
	Rank 6	Rank 7	Rank 8	Rank 9	Rank 10
	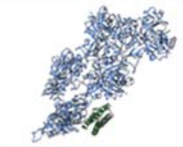	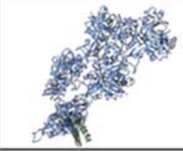	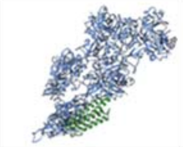	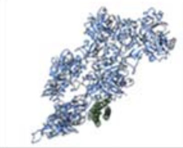	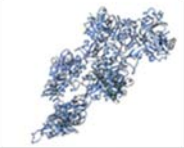
Docking Score	-161.81	-159.52	-159.34	-157.16	-153.63
Confidence Score	0.5588	0.5475	0.5466	0.5357	0.5181
Ligand rmsd (Å)	58.69	104.87	129.43	101.75	75.35

*The models are ranked according to the docking scores.
*The ligand RMSDs from the input structures or modeled structures by homology modeling.

Figure S3 Top 10 solutions of Hdock server docking results for F-Actin filaments and IMP/TLNRD1/TLNRD1_4H. The top 10 binding models were proposed for IMP (red)/Actin, TLNRD1 (light green)/Actin and TLNRD1_4H (dark green)/Actin by HDOCK server, each with Docking Score and Confidence Score indicated.

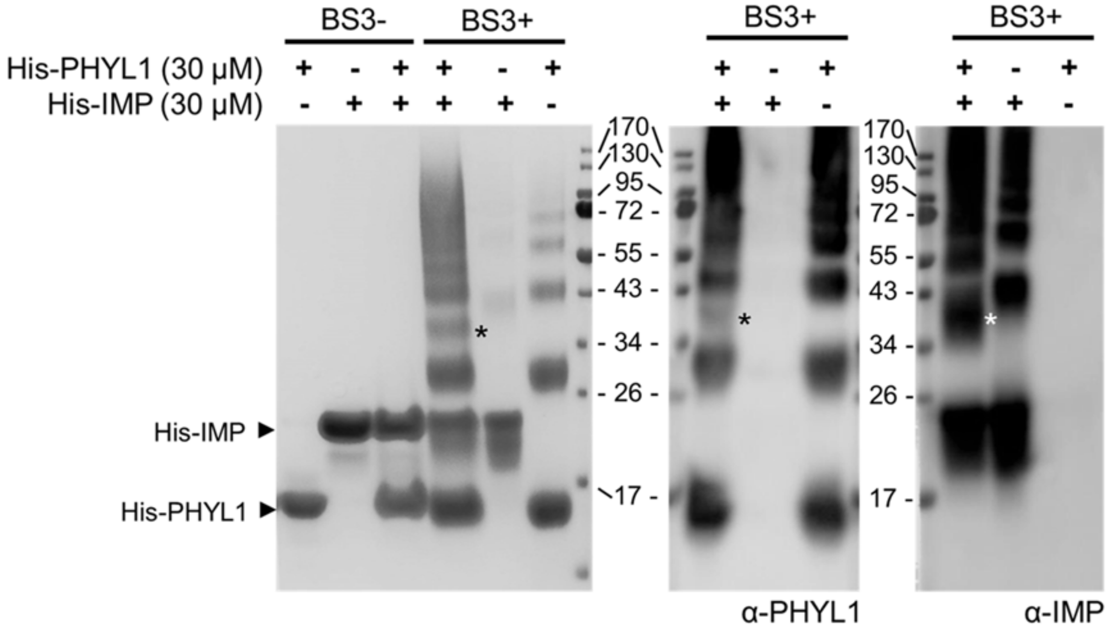

Figure S4 Cross-linking analysis and western blot assay to confirm the binding of IMP and PHYL1. Cross-linking analysis of PHYL1 and IMP. Shifting band due to protein interaction was marked with an asterisk. Both anti-PHYL1 and anti-IMP antibodies were diluted 1:5000 for detection.

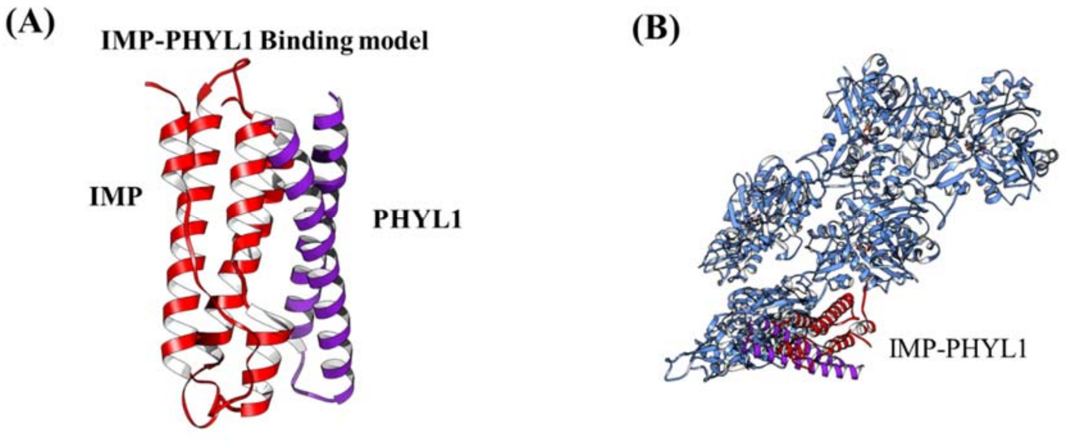

Figure S5 Structural analysis modelling of IMP, PHYL1 and F-Actin. (A) The proposed IMP/PHYL1 binding model by HDOCK server. Docking Score, -199.51; Confidence Score, 0.729. (B) The proposed IMP/PHYL1-Actin binding model by HDOCK server with Docking Score, -198.65 and Confidence Score, 0.726. The F-Actin model is coloured in blue, the IMP model is coloured in red while the PHYL1 model is coloured in purple.
